# Study on the relationship between sleep–wake states and prognosis in patients with intracranial arterial stenosis and ischemic stroke

**DOI:** 10.3389/fneur.2025.1531241

**Published:** 2025-06-30

**Authors:** Xiaodong Yuan, Jing Xue, Yongshan Fu, Ya Ou, Ying Zhao, Cuiping Yan, Pingshu Zhang

**Affiliations:** ^1^Department of Neurology, Kailuan General Hospital, North China University of Science and Technology, Tangshan, China; ^2^Key Laboratory of Neurobiological Function, Tangshan, China

**Keywords:** intracranial arterial stenosis, ischemic stroke, sleep–wake state, prognosis, prognostic factors analysis

## Abstract

**Objective:**

To evaluate the influence of the sleep–wake state on the prognosis of patients with ischemic stroke.

**Methods:**

Consecutive patients with intracranial ischemic stroke due to arterial stenosis were included (198 cases). The control group consisted of contemporaneous patients without cerebrovascular stenosis or any diagnosed cerebrovascular disease (77 cases). Collect the following variables of the patients, including the total recording time during the day and night, total sleep time, sleep latency, rapid eye movement (REM) sleep latency, wake time after falling asleep, light sleep stage (N1, N2 stage), deep sleep stage (N3 stage), and non-rapid eye movement (non-rapid eye movement) (NREM) sleep stage, rapid eye movement (REM) sleep stage, and stroke topography (anterior circulation and posterior circulation ischemic stroke). The primary outcome was the functional status at discharge, evaluated using the modified Rankin Scale (mRS): good prognosis (mRS ≤ 2) and poor prognosis (mRS > 2).

**Results:**

In the regression analysis of prognostic influencing factors in patients with ACIS, it was concluded that an increase in daytime deep sleep time was associated with an increased possibility of adverse outcomes in patients with ACIS (*OR* = 1.026; 95% *CI*, 1.003–1.048, *p* = 0.024). In the regression analysis of prognostic influencing factors in patients with PCIS, it was concluded that during PCIS, the duration of deep sleep was longer (*OR* = 1.038; 95% *CI*, 1.001–1.077, *p* = 0.046) and the duration of nocturnal NREM staging was longer (*OR* = 1.010; patients with 95% *CI*, 1.000–1.020, *p* = 0.042) had a higher possibility of adverse outcomes.

**Conclusion:**

The sleep–wake state of patients with intracranial artery stenoischemic stroke changes. The main characteristics are increased diurnal sleep, increased incidence of daytime sleep, and disordered sleep–wake phases. In patients with ACIS, the diurnal sleep–wake biological rhythm mainly characterized by poor daytime stability is unbalanced. The longer the duration of daytime deep sleep and nighttime NREM sleep, the higher the possibility of adverse outcomes in patients with intracranial artery stenotic ischemic stroke.

## Highlights

- Patients with ischemic stroke exhibit changes in sleep–wake states.
- Daytime deep sleep is a prognostic factor in patients with ischemic stroke.
- Nighttime NREM sleep is a prognostic factor in patients with ischemic stroke.

## 1 Introduction

Acute ischemic stroke (AIS) can be classified based on the ischemic site into acute anterior circulation ischemic stroke and acute posterior circulation ischemic stroke, with the former being the most prevalent, accounting for 80–90% of all stroke cases (1). Patients with posterior circulation ischemic stroke generally have a poor prognosis, especially those with moderate to severe clinical manifestations of basilar artery occlusion, where mortality and disability rates are as high as 80%, significantly impacting the health index of the population in China (2, 3). It is well known that stable sleep is related to body metabolism and temperature regulation and plays a crucial role in modulating inflammation and apoptosis, thereby enhancing neuroprotective mechanisms and promoting neuroplasticity, which is fundamental for neurological recovery post-stroke (4, 5). Several studies (6–8) have analyzed sleep–wake phase changes following stroke in humans and rodents, characterized by increased NREM1-3 sleep, elevated arousal index, and persistent sleep–wake disturbances (9–11). Changes in the sleep–wake phases during stroke recovery are fundamental to neuroplasticity and can be used to monitor and evaluate functional recovery post-stroke by tracking these changes (12).

Studies have shown that there are certain differences between anterior circulation and posterior circulation in terms of anatomical structure, risk factors, clinical manifestations and etiology of stroke. Therefore, the outcomes of the two are considered different (13). The incidence of stroke patients with intracranial artery stenosis is high and the prognosis is poor. Therefore, patients with this disease are selected as the research subjects. It is assumed that the sleep–wake state of stroke patients with intracranial artery stenosis changes significantly. It is expected that this change will affect the prognosis and recovery of neurological function, providing an objective basis for the formulation of clinical prognosis treatment plans.

## 2 Materials and methods

### 2.1 Study subjects

**Figure 1:**
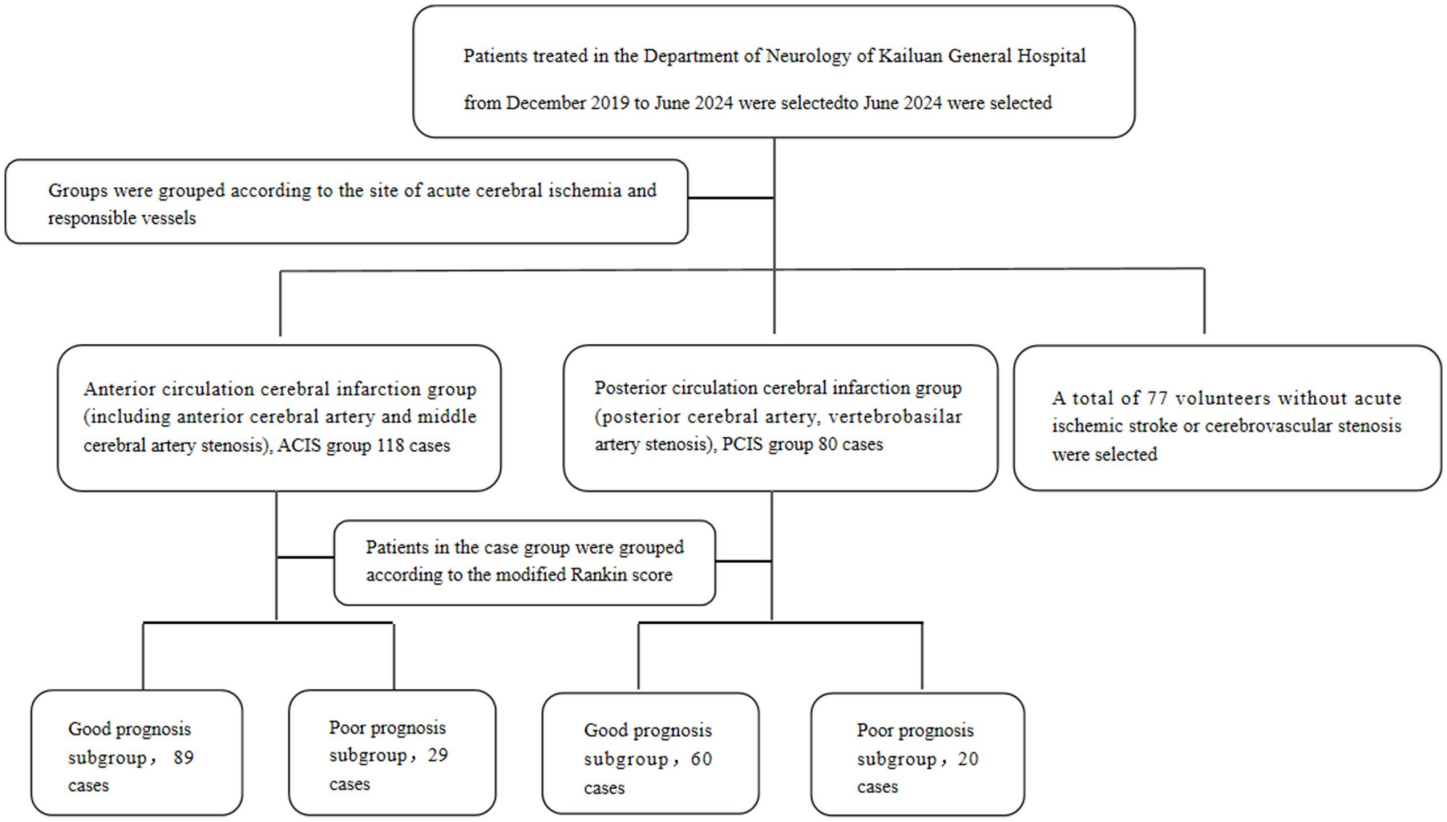
Grouping flowchart.

Patients with acute ischemic stroke treated at the Department of Neurology, Kailuan General Hospital affiliated with North China University of Science and Technology, between December 2019 and June 2024, were selected as study subjects. According to the ischemic sites and responsible vessels of patients with intracranial arterial stenotic ischemic stroke, patients were divided into two groups: the anterior circulation ischemic stroke (ACIS) group, which includes patients with anterior cerebral artery and middle cerebral artery stenosis; and the posterior circulation ischemic stroke (PCIS) group, which includes patients with posterior cerebral artery and vertebrobasilar artery stenosis, see Figure 1 for details.

Inclusion criteria:

1. All selected patients met the diagnostic criteria of the “2021 Guidelines for the Prevention of Stroke in Patients with Stroke and Transient Ischemic Attack: A Guideline from the American Heart Association/American Stroke Association” (14).
2. Confirmed by transcranial magnetic resonance imaging (MRI, GE Discovery MR750W), with lesions distributed in the anterior cerebral artery, middle cerebral artery, frontal and parietal lobes, centrum semiovale, basal ganglia, insula, and hippocampus. Magnetic resonance angiography (MRA, GE Discovery MR750W) showed stenosis or occlusion of the anterior cerebral artery or middle cerebral artery, or color Doppler ultrasonography detected a peak systolic velocity (PSV) in the middle cerebral artery ≥140 cm/s (mild stenosis: 140 cm/s ≤ PSV < 180 cm/s, moderate stenosis: 180 cm/s ≤ PSV < 220 cm/s, severe stenosis: PSV ≥ 220 cm/s) (14). This is the specific inclusion criterion for the ACIS group.
3. Confirmed by transcranial MRI, with lesions mainly distributed in the thalamus, pons, medulla, cerebellum, and corpus callosum supplied by the posterior cerebral artery and vertebrobasilar artery, with MRA showing stenosis or occlusion of these arteries. This is the specific inclusion criterion for the PCIS group.
4. Unconscious disorder. According to the Glasgow coma scale (GCS), a GCS score of 15 points was used to determine unconsciousness disorder. All patients with a GCS score lower than 15 points were considered to have consciousness disorder. GCS consists of three items: eye-opening response (with a score range of 1 to 4 points), verbal response (with a score range of 1 to 5 points), and motor response (with a score range of 1 to 6 points), with a total score range of 3 to 15 points.
5. Total sleep monitoring time during both day and night ≥ 5 days, with a total recording time of no less than 8 h per day.

Exclusion criteria:

1. History of psychiatric disorders such as anxiety, depression, epilepsy, etc.
2. Severe functional impairment of major organs (heart, lung, liver, kidney) or malignant tumors.
3. Use of sedative or sleep-inducing medications.
4. History of other neurological disorders: neuromuscular junction and muscle diseases, neurodegenerative diseases.
5. Inability to cooperate with the examination due to other severe physical illnesses.

Patients who underwent MRI and MRA examinations during the same period and whose results showed no cerebrovascular diseases or cerebrovascular stenosis were selected as the control group. Volunteers with good compliance met the conditions of sleep monitoring day, total sleep monitoring time at night ≥5, total recording time in 1 day no less than 8 h, and the age range and gender proportion were matched with the case group. Exclusion criteria were the same as those for the ACIS and PCIS groups.

The study included 118 subjects in the ACIS group (age 35–90 years), 80 subjects in the PCIS group (age 34–92 years), and 77 subjects in the control group (age 56–89 years). This study complies with the Declaration of Helsinki and has been approved by the Medical Ethics Committee of Kailuan General Hospital affiliated with the North China University of Science and Technology (approval number 2023005). All participants or their legal representatives signed informed consent forms.

### 2.2 Methods

#### 2.2.1 Clinical data collection

Clinical data collection includes gender, age, body mass index (BMI), history of hypertension, diabetes, hyperhomocysteinemia, hyperlipidemia, smoking, and alcohol consumption.

#### 2.2.2 Sleep parameter monitoring

**Figure 2:**
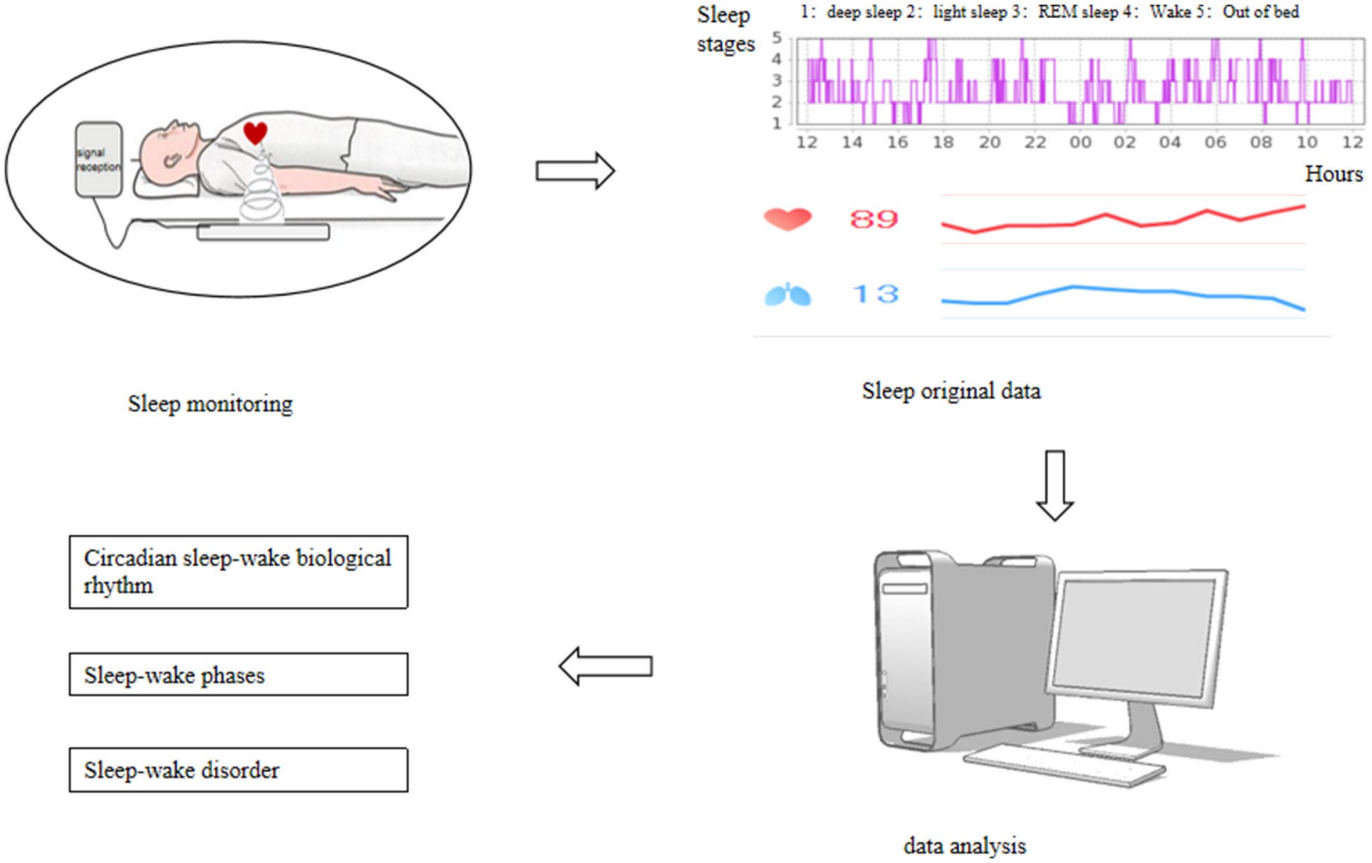
Sleep monitoring procedure.

Sleep parameters were monitored using the SC-500 sleep monitor produced by Nanjing Boyichuan Haiyun Electronic Technology Co., Ltd. Sleep parameters were recorded for the periods 6:00–18:00 and 18:00–6:00 the following day. The parameters recorded include total recording time, total sleep time, sleep latency, rapid eye movement (REM) sleep latency, wake time after sleep onset, light sleep stages (N1, N2), deep sleep stage (N3), non-rapid eye movement (NREM) sleep, and REM sleep. Each parameter was averaged over 5 days, and data with a 24 h recording time of less than 8 h were excluded. See Figure 2 for details.

#### 2.2.3 Criteria for determining circadian sleep–wake rhythm

The circadian rhythm was assessed using inter-daily stability (IS) and intra-daily variability (IV) to evaluate changes in the body’s circadian rhythm (15, 16). IS assesses the stability of 24 h day-night activity variation and the balance between rest-activity rhythms and circadian changes. Its value ranges from 0 to 1, with values closer to 1 indicating a more stable circadian rhythm. IV evaluates the fragmentation of day-night activity variation by calculating the frequency and degree of rest-activity transitions within a given period. Its value also ranges from 0 to 1, with higher values indicating more fragmented circadian rhythms.

The calculation is based on heart rate indicators monitored during sleep. The specific formulas are as follows:

$$\text{IS}=\frac{n\;\sum_{h=1}^{p}{\left({\bar{x}}_{h}-\bar{x}\right)}^{2}}{p\;\sum_{i=1}^{n}{\left(x_{i}-\bar{x}\right)}^{2}}\;\mathrm{IV}=\frac{n\;\sum_{i=2}^{n}{\left(x_{i}-x_{i-1}\right)}^{2}}{(n-1)\;\sum_{i=1}^{n}{\left(x_{i}-\bar{x}\right)}^{2}}$$

*n*: Total monitoring time for heart rate (in hours); p: 24 h; ${\bar{x}}_{h}$ : Average heart rate during the h-th hour of daily monitoring; $\bar{x}$ : Average hourly heart rate during the monitoring period; ${\bar{x}}_{i}$ : Average heart rate during the i-th hour of the monitoring period.

#### 2.2.4 Criteria for determining sleep–wake disorders

Sleep–wake disorders were assessed using various sleep parameters obtained from sleep monitoring to interpret the day and night circadian rhythm results (16, 17).

1. Increased total sleep time post-stroke: total sleep time > 10 h/day.
2. Reversed sleep cycle post-stroke: daytime total sleep time > nighttime total sleep time.
3. Increased daytime sleep post-stroke: daytime total sleep time > 6 h/day.
4. Nighttime sleep maintenance disorder: nighttime total sleep time < 5 h/night.
5. Difficulty falling asleep: nighttime sleep latency > 30 min.
6. Low sleep efficiency: nighttime sleep efficiency < 75%.

### 2.3 Statistical methods

Statistical analysis was performed using SPSS 26.0 software. Measurement data conforming to a normal distribution are expressed as mean ± standard deviation ( $\bar{x}$ ±s). Comparisons among multiple groups were conducted using one-way analysis of variance (ANOVA), and pairwise comparisons between groups were performed using the q-test. Non-normally distributed data are expressed as median (*P*25, *P*75), and comparisons among multiple groups were conducted using the Kruskal-Wallis H test, with pairwise comparisons between groups performed using non-parametric tests. Count data are expressed as frequency (%), and comparisons among multiple groups were conducted using the chi-square test (χ^2^), with pairwise comparisons between groups performed using the Bonferroni test. Multivariate logistic regression analysis was used to investigate factors influencing stroke prognosis. A *p* value of less than 0.05 was considered statistically significant.

## 3 Results

### 3.1 Comparison of baseline data among the three groups

The study subjects were divided into three groups based on inclusion and exclusion criteria: the ACIS group, the PCIS group, and the control group. The ACIS group included 118 patients with an average age of 67.42 ± 9.90 years, 61.86% of whom were male. The PCIS group included 80 patients with an average age of 66.19 ± 10.47 years, 67.5% of whom were male. The control group included 77 volunteers with an average age of 65.97 ± 8.06 years, 63.64% of whom were male.

Compared to the control group, the ACIS group had a higher proportion of hyperhomocysteinemia, as did the PCIS group. Both the ACIS and PCIS groups had higher proportions of higher rates of hypertension and diabetes. Compared to the ACIS group, the PCIS group had a higher proportion of hyperhomocysteinemia. All differences were statistically significant (*p* < 0.01), as shown in Table 1.

**Table 1:** Comparison of baseline data among the three groups.

Variables	Control group (*n* = 77)	ACIS group (*n* = 118)	PCIS group (*n* = 80)	H/χ2 value	*P*-value
Gender (male/female)	49/28	73/45	54/26	0.663	0.718
Age, years	65.97 ± 8.06	67.42 ± 9.90	66.19 ± 10.47	3.922	0.141
BMI, kg/m^2^	24.84 (22.51, 26.45)	24.73 (23.10, 26.17)	24.41 (23.09, 25.61)	0.048	0.976
Hypertension, *n* (%)	31 (40.30)	100 (84.79)^a^	71 (88.80)^b^	60.827	<0.001
Diabetes, *n* (%)	10 (13.00)	65 (55.10)^a^	39 (48.8)^b^	36.499	<0.001
Hyperlipidemian, (%)	5 (6.50)	21 (17.80)	11 (13.80)	5.121	0.077
Hyperlipidemia, *n* (%)	7 (9.10)	26 (22.00)^a^	22 (27.50)^c^	8.845	0.012
Smoking, *n* (%)	25 (32.50)	50 (42.40)	30 (37.50)	1.959	0.375
Alcohol consumption, *n* (%)	21 (27.30)	45 (38.10)	27 (33.80)	2.457	0.293

a Represents the comparison between the ACIS group and the control group, *P* < 0.05.

b Represents the comparison between the PCIS group and the control group, *P* < 0.05.

c Represents the comparison between the PCIS group and the ACIS group, *P* < 0.05.

### 3.2 Comparison of circadian sleep–wake rhythms among three groups

Compared to the control group, the ACIS group exhibited poorer daytime stability. In contrast, the PCIS group showed more stable daytime stability compared to the ACIS group (*p* = 0.007), as shown in Table 2.

**Table 2:** Comparison of circadian sleep–wake rhythms among three groups.

Variables	Control group (*n* = 77)	ACIS group (*n* = 118)	PCIS group (*n* = 80)	*H* value	*p* value
IS	0.49 (0.32, 0.66)	0.40 (0.24, 0.56)^a^	0.48 (0.34, 0.67)^b^	11.656	0.003
IV	0.80 (0.65, 0.92)	0.76 (0.64, 0.89)	0.75 (0.49, 0.98)	0.084	0.959

a Represents the comparison between the ACIS group and control group, *P* < 0.05.

b Represents the comparison between the PCIS group and the control group, *P* < 0.05.

### 3.3 Comparison of daytime sleep–wake phases among three groups

Compared to the control group, the ACIS group had an increased wake time after sleep onset (*p* = 0.005). Both the ACIS and PCIS groups had increases in total daytime sleep time, light sleep phase, deep sleep phase, NREM sleep phase, and REM sleep phase, with all *p* values < 0.001, as shown in Table 3.

**Table 3:** Comparison of daytime sleep–wake phases among three groups.

Variables	Control group (*n* = 77)	ACIS group (*n* = 118)	PCIS group (*n* = 80)	*H* value	*P* value
Total sleep time, min	205.20 (157.10, 278.30)	299.60 (233.30, 416.35)^a^	300.40 (216.75, 423.25)^b^	39.158	<0.001
sleep latency, min	4.80 (2.40, 8.00)	4.10 (1.15, 9.65)	5.60 (3.25, 9.00)	4.129	0.127
REM sleep latency, min	42.80 (25.60, 60.10)	39.40 (24.75, 52.45)	40.90 (25.25, 57.00)	1.179	0.555
wake time after sleep onset, min	61.40 (44.30, 82.20)	74.90 (60.15, 96.65)^a^	72.90 (56.20, 94.55)	10.618	0.005
light sleep stages (N1-N2 stages), min	160.80 (123.20, 226.50)	234.90 (167.25, 300.45)^a^	216.90 (158.10, 296.00)^b^	25.157	<0.001
Deep sleep stages (N3 stages), min	8.00 (4.00, 13.80)	17.10 (8.15, 26.30)^a^	13.20 (7.15, 28.95)^b^	27.834	<0.001
NREM sleep, min	171.60 (131.30, 238.40)	248.10 (180.95, 333.70)^a^	233.50 (175.90, 312.80)^b^	28.738	<0.001
REM sleep, min	31.00 (20.40, 46.80)	56.50 (29.45, 100.15)^a^	63.60 (32.25, 89.90)^b^	36.319	<0.001

a Represents the comparison between ACIS group and control group, *P* < 0.05.

b Represents the comparison between PCIS group and control group, *P* < 0.05.

### 3.4 Comparison of nighttime sleep–wake phases among three groups

Compared to the control group, both the ACIS and PCIS groups had increases in total nighttime sleep time, sleep latency, light sleep phase, NREM sleep phase, and REM sleep phase, with *p* values of < 0.001, 0.003, < 0.001, < 0.001, and 0.001, respectively. Compared to the PCIS group, the ACIS group had a higher NREM sleep (*p* < 0.001), as shown in Table 4.

**Table 4:** Comparison of nighttime sleep–wake rhythm phases among three groups.

Variables	Control group (*n* = 77)	ACIS group (*n* = 118)	PCIS group (*n* = 80)	*F/Z* value	*P* value
Total sleep time (min)	471.00 (424.50, 518.00)	516.60 (459.60, 570.20)^a^	527.00 (456.45, 568.45)^b^	24.049	<0.001
sleep latency (min)	11.00 (6.10, 17.60)	7.70 (3.00, 15.45)^a^	11.10 (3.50, 20.75)^b^	8.149	0.017
REM sleep latency (min)	55.40 (42.00, 78.50)	47.50 (31.70, 73.00)	50.80 (34.15, 66.00)	4.657	0.097
wake time after sleep onset (min)	76.60 (64.00, 97.60)	84.00 (68.05, 102.65)	79.80 (65.20, 96.15)	3.257	0.196
light sleep stages (N1-N2 stages) (min)	301.75 ± 51.96	330.61 ± 52.84^a^	331.42 ± 50.54^b^	11.106	<0.001
Deep sleep stages (N3 stages) (min)	63.00 (44.30, 80.20)	60.50 (46.45, 81.15)	59.60 (45.90, 77.15)	0.635	0.728
NREM sleep (min)	372.40 (336.90, 397.40)	401.30 (367.90, 441.20)^a^	376.50 (327.60, 422.10)^c^	19.358	<0.001
REM sleep (min)	98.99 ± 26.86	111.06 ± 30.60^a^	112.10 ± 26.47^b^	6.898	0.001

a Represents the comparison between the ACIS group and the control group, *P* < 0.05.

b Represents the comparison between the PCIS group and the control group, *P* < 0.05.

c Represents the comparison between the PCIS group and the ACIS group, *P* < 0.05.

### 3.5 Comparison of sleep–wake disorders among three groups

Compared to the control group, the ACIS group had a higher proportion of low sleep efficiency. Both the ACIS and PCIS groups showed increased rates of excessive daytime sleep and increased occurrence of daytime sleep (*p* = 0.003 and < 0.001, respectively). Compared to the ACIS group, the PCIS group had no patients with low sleep efficiency (*p* = 0.003), as shown in Table 5.

**Table 5:** Comparison of circadian sleep–wake rhythm between the three groups.

Variables	Control group (*n* = 77)	ACIS group (*n* = 118)	PCIS group (*n* = 80)	*χ2* value	*P* value
Increased total sleep time, *n* (%)	60 (77.90)	109 (92.40)^a^	74 (92.50)^b^	11.340	0.003
Reversed sleep cycle, *n* (%)	1 (1.30)	6 (5.10)	5 (6.30)	2.562	0.278
Increased daytime sleep, *n* (%)	8 (10.40)	43 (36.40)^a^	33 (41.30)^b^	20.999	<0.001
Nighttime sleep maintenance disorder, *n* (%)	3 (3.90)	2 (1.70)	0 (0.00)	3.354	0.187
Difficulty falling asleep, *n* (%)	7 (9.10)	6 (5.10)	5 (6.30)	2.635	0.268
Low sleep efficiency, *n* (%)	0 (0.00)	8 (6.80)^a^	0 (0.00)^c^	10.963	0.003

a Represents the comparison between the ACIS group and the control group, *P* < 0.05.

b Represents the comparison between the PCIS group and the control group, *P* < 0.05.

c Represents the comparison between the PCIS group and the ACIS group, *P* < 0.05.

### 3.6 Factors influencing prognosis in ACIS patients

The prognosis of patients was assessed using the modified Rankin Scale (mRS). mRS Score: 0 points, completely asymptomatic; 1 point, mild symptoms but not affecting normal life; 2 points, mild disability but still able to take care of daily life independently; 3 points, severe disability, requiring partial assistance to complete daily life; 4 points, severe disability, requiring assistance to complete daily life; 5 points. Severely disabled and completely dependent on assistance in daily life. Patients were divided into subgroups based on their mRS scores at discharge. In the ACIS group, there were 89 patients with a good prognosis (mRS score ≤ 2) and 29 patients with a poor prognosis (mRS score > 2). In the PCIS group, there were 80 patients with a good prognosis (mRS score ≤ 2) and 20 patients with a poor prognosis (mRS score > 2). For the prognostic analysis of ACIS patients, the prognosis was set as the dependent variable (assignment: good prognosis = 0, poor prognosis = 1). Variables that were significant in univariate analysis and had no collinearity were selected as independent variables, including hypertension (assignment: no = 0, yes = 1), diabetes (assignment: no = 0, yes = 1), hyperhomocysteinemia (assignment: no = 0, yes = 1), IS (measured value), daytime total sleep time (measured value), daytime wake time after sleep onset (measured value), daytime light sleep stage (measured value), daytime deep sleep stage (measured value), daytime NREM sleep stage (measured value), daytime REM sleep stage (measured value), nighttime total sleep time (measured value), nighttime sleep latency (measured value), nighttime light sleep stage (measured value), nighttime NREM sleep stage (measured value), nighttime REM sleep stage (measured value), increased day and night sleep (assignment: no = 0, yes = 1), increased daytime sleep (assignment: no = 0, yes = 1), and reduced sleep efficiency (assignment: no = 0, yes = 1). A multivariate logistic stepwise regression analysis was performed. The results showed that increased daytime deep sleep time is associated with an increased possibility of adverse outcomes in patients with ACIS (*OR* = 1.026) 95% *CI*, 1.003–1.048, *p* = 0.024, as shown in Table 6.

**Table 6:** Multivariate logistic regression analysis of prognostic factors in ACIS patients.

Variables	B	BE	Waldχ*2*	*P* value	*OR* value (95%*CI*)
Daytime deep sleep stage (N3 stage)	0.025	0.011	5.072	0.024	1.026 (1.003–1.048)
constant	−1.703	0.345	24.316	<0.001	-

### 3.7 Prognostic factors for PCIS patients

For the prognostic analysis of PCIS patients, the prognosis was set as the dependent variable (assignment: good prognosis = 0, poor prognosis = 1). Variables that were significant in univariate analysis and had no collinearity were selected as independent variables, including hypertension (assignment: no = 0, yes = 1), diabetes (assignment: no = 0, yes = 1), hyperhomocysteinemia (assignment: no = 0, yes = 1), IS (measured value), daytime total sleep time (measured value), daytime wake time after sleep onset (measured value), daytime light sleep stage (measured value), daytime deep sleep stage (measured value), daytime NREM sleep stage (measured value), daytime REM sleep stage (measured value), nighttime total sleep time (measured value), nighttime sleep latency (measured value), nighttime light sleep stage (measured value), nighttime NREM sleep stage (measured value), nighttime REM sleep stage (measured value), increased day and night sleep (assignment: no = 0, yes = 1), increased daytime sleep (assignment: no = 0, yes = 1), and reduced sleep efficiency (assignment: no = 0, yes = 1). A multivariate logistic stepwise regression analysis was conducted, and the results showed that in PCIS, the duration of deep sleep was longer (*OR* = 1.038; 95% *CI*, 1.001–1.077, *p* = 0.046) and the duration of nocturnal NREM staging was longer (*OR* = 1.010; patients with 95% *CI*, 1.000–1.020, *p* = 0.042) were more likely to have adverse outcomes, as shown in Table 7.

**Table 7:** Multivariate logistic regression analysis of prognostic factors in PCIS patients.

Variables	B	BE	Waldχ*2*	*P* value	*OR* value (95%*CI*)
Daytime deep sleep stage (N3 stage)	0.037	0.019	3.979	0.046	1.038 (1.001–1.077)
Nighttime NREM stage	0.010	0.005	4.149	0.042	1.010 (1.000–1.020)
Constant	−5.573	1.973	7.978	0.005	–

## 4 Discussion

Our study results indicate that the prevalence of sleep–wake disturbances is high in both ACIS and PCIS patients. The main manifestation is a generalized increase in sleep duration across various phases. ACIS patients primarily exhibit daytime instability leading to a disrupted circadian sleep–wake rhythm. The longer the duration of daytime deep sleep and nighttime NREM sleep, the higher the possibility of adverse outcomes in patients with intracranial artery stenotic ischemic stroke.

In the quantitative assessment of sleep–wake disturbances, we found that total sleep time, light sleep phase, NREM sleep phase, and REM sleep phase were all increased in both ACIS and PCIS patients. Daytime sleep–wake phase including deep sleep phase and nighttime sleep–wake phase including sleep latency in ACIS patients were higher than those in control group. PCIS patients showed increased wake time after sleep onset (WASO), and deep sleep phase compared to controls. These findings suggest a general increase in sleep duration in both ACIS and PCIS patients. The increase in sleep duration is associated with a higher risk of overall ischemic stroke, particularly in the elderly. This study is consistent with previous studies (18, 19). Sleep–wake phases include NREM sleep, REM sleep, and wakefulness. NREM sleep consists of light sleep and deep sleep. Sleep–wake behavior is vital for maintaining overall health, and supporting functions such as learning, memory consolidation, energy restoration, immunity enhancement, and growth development. The classical arousal regulation theory is based on the ascending reticular activating system (ARAS), which includes the dorsal and ventral pathways that relay activity from the brainstem to the cerebral cortex, promoting and maintaining wakefulness through noradrenergic neurons in the locus coeruleus, serotonergic neurons in the dorsal raphe nucleus, and thalamic paraventricular nucleus projections (20). These two major neural circuits coordinate with each other to jointly complete the transition between sleep and wakefulness. The physiological mechanism by which ischemic and hypoxic damage near the lesion after stroke interferes with sleep–wake is that neuronal populations such as the basal ganglia prebrain, thalamus, hypothalamus, pons, and medulla oblongata in the brain specifically inhibit or promote the sleep–wake cycle. Neural networks are formed through synaptic connections among the nuclei to initiate and maintain the wake, NREM phase, and REM phase (21). The maintenance and transition of the sleep–wake cycle depend on the dynamic changes of different neurotransmitter levels, and different stroke sites can affect the secretion of different neurotransmitters, thereby manifested as abnormalities in different sleep phases.

Our study also found that ACIS patients exhibited poorer daytime stability compared to PCIS patients, with disrupted circadian rhythms. PCIS patients showed more stable daytime coordination and less circadian variability. This suggests that compared to PCIS patients, ACIS patients have reduced sensitivity to light, impaired circadian recognition, and shorter wakefulness durations. The circadian system synchronizes the body’s internal states with external cues like light, maintaining physiological and behavioral rhythms. On a molecular level, the circadian system involves a transcription-translation feedback loop characterized by 24 h oscillations influenced by clock genes (primarily clock and BMAL1 genes) and their protein products. The central pacemaker, located in the suprachiasmatic nucleus (SCN), regulates circadian rhythms through light–dark cycles, endogenous metabolism, and hormone levels (22). The SCN projects other hypothalamic nuclei, particularly the subparaventricular zone (SPZ), dorsomedial hypothalamus (DMH), and ventromedial hypothalamus (VMH). The SCN integrates molecular timing with neuronal firing rhythms, with SCN firing rates peaking during light periods and SPZ firing rates during dark periods. The SPZ acts as a key relay for circadian rhythms, promoting wakefulness during active periods and sleep during rest periods (23). Experimental stroke models have shown that acute cerebral ischemia disrupts circadian stability, leading to phase shifts and sleep–wake imbalances, especially when the stroke affects anterior circulation areas, causing SCN dysfunction and circadian disruption (24). Patients with ACIS have disrupted the regulation of circadian rhythms and have a higher degree of sleep–wake instability.

We further found that in the regression analysis of prognostic influencing factors in patients with intracranial artery stenotic ischemic stroke, it was concluded that the longer the daytime deep sleep period, the greater the possibility of adverse prognostic events in patients with ACIS and those with PCIS, with *OR*s of 1.009 (95% *CI*, 1.003–1.058) and 1.038 (95% *CI*, 1.001–1.077), respectively. Deep sleep, as part of SWS, during SWS, cerebral blood flow decreases by 25% and cerebral blood volume (CBV) drops by approximately 10%, allowing cerebrospinal fluid to flow into the third and fourth ventricles, promoting communication between fluid chambers and waste clearance. At the same time, the cortex is isolated from the environment, and the cell nuclei that transmit signals to the outside lose response to the outside. This leads to a reduction in oxygen metabolism in the brain, but the neocortex in the brain is active. The reactivity of cortical neurons to the corpus callosum is enhanced, and neurons form coherent sets, which is conducive to the long-term consolidation of new memories (25). Post-stroke neuronal excitation induces rhythmic, high-amplitude waves resembling SWS oscillations, which enhance axonal sprouting in ischemic cortical circuits, aiding brain plasticity and recovery (26). Additionally, SWS oscillations strengthen synaptic connections, maintaining energy levels and neuronal selectivity, crucial for post-stroke sensorimotor recovery (27).

We also found that the longer the NREM sleep period at night, the greater the possibility of poor prognosis in patients with PCIS, with an *OR* of 1.010 (95% *CI*, 1.000–1.020). NREM sleep includes deep and light sleep, suggesting that PCIS patients have more deep sleep over 24 h compared to ACIS patients. Previous studies have shown that PCIS patients experience increased deep sleep and wakefulness (28). This is because the thalamus, a key arousal regulation center in the posterior circulation, when infarcted, leads to decreased sleep quality and increased sleep–wake disturbances, extending total sleep time during the day (29, 30). The pons, commonly affected in posterior circulation strokes, includes the ARAS and is sensitive to ischemia, leading to disrupted neural activity, decreased brain network connectivity, and altered wakefulness states, affecting overall cognitive function and causing Wallerian degeneration in distant neural fibers, exacerbating motor and speech deficits. Overall, deep sleep directly promotes neural circuit plasticity during sleep, serving as an intervention window for stroke recovery. Identifying and managing risk factors early can reduce stroke risk and improve prognosis by mitigating adverse events.

Our study has several strengths. Firstly, our target population was specifically categorized based on ischemic locations and responsible vessel stenosis, providing a comprehensive examination of sleep–wake changes and prognostic factors in intracranial arterial stenosis ischemic stroke patients. Secondly, our sleep data collection was reliable, with 5 days of monitored sleep data divided into day and night cycles, averaging each parameter. Thirdly, our findings are clinically relevant, offering valuable insights for patient management and prognosis assessment.

## 5 Limitations

This study has several limitations. Firstly, the sample size is relatively small and region-specific, with a single-source sample. Future studies should aim to increase the sample size and include samples from different regions. Secondly, there is a lack of multi-level analysis regarding the degree of cerebrovascular stenosis in the study population. Thirdly, there was no follow-up study to track the prognosis of the participants. Fourth, this study has potential confounding variables (for example, infarction area/location, sedative use, unmeasured comorbidities), selection bias in the control group, and the lack of longitudinal follow-up after discharge.

## 6 Conclusion

Patients with intracranial arterial stenosis ischemic stroke exhibit changes in sleep–wake patterns, characterized by increased total sleep duration, higher daytime sleep incidence, and disrupted sleep–wake phases. ACIS patients, in particular, show daytime instability, leading to an imbalanced circadian sleep–wake rhythm. Daytime deep sleep and nighttime NREM sleep are identified as prognostic factors for these patients.

## Data Availability

The original contributions presented in the study are included in the article/supplementary material, further inquiries can be directed to the corresponding authors.
